# Association between syndecan-4 and subclinical atherosclerosis in ankylosing spondylitis

**DOI:** 10.1097/MD.0000000000037019

**Published:** 2024-01-19

**Authors:** Ahmet L. Sertdemir, Ahmet T. Şahin, Mustafa Duran, Mustafa Çelik, Sefa Tatar, İrem Oktay, Yakup Alsancak

**Affiliations:** a Department of Cardiology, Meram School of Medicine, Necmettin Erbakan University, Konya, Turkey; b Department of Cardiology, Konya City Hospital, Konya, Turkey.

**Keywords:** ankylosing spondylitis, subclinical atherosclerosis, syndecan-4

## Abstract

**Background:**

Despite advances in the diagnosis and treatment of ankylosing spondylitis (AS), the risk of cardiovascular complications in AS patients is still higher than in the general population. Macrophages are at the intersection of the basic pathogenetic processes of AS and atherosclerosis. Although syndecan-4 (SDC4) mediates a variety of biological processes, the role of SDC4 in macrophage-mediated atherogenesis in AS patients remains unclear. Herein, we aimed to investigate the role of SDC4 in subclinical atherosclerosis in AS patients.

**Methods:**

Subjects were selected from eligible AS patients and control subjects without a prior history of AS who were referred to the rheumatology outpatient clinics. All participants’ past medical records and clinical, and demographic characteristics were scanned. In addition, carotid intima-media thickness (CIMT) measurement and disease activity index measurement were applied to all patients.

**Results:**

According to our data, serum SDC4 level was significantly higher among AS patients compared with the control group (6.7 [1.5–35.0] ng/mL vs 5.1 [0.1–12.5] ng/mL, *P* < .001). The calculated CIMT was also significantly higher in AS patients than in the control group (0.6 [0.3–0.9] mm vs 0.4 (0.2–0.7), *P* < .001]. Additionally, serum C-reactive protein level and SDC4 level were independent predictors of AS and strongly associated with CIMT. Linear regression analysis showed that serum SDC4 level was the best predictor of CIMT (*P* = .004).

**Conclusion:**

Our data indicate that serum SDC4 levels provide comprehensive information about the clinical activity of the disease and subclinical atherosclerosis in AS patients.

## 1. Introduction

Ankylosing spondylitis (AS) is a chronic, progressive, and inflammatory disease of the axial skeleton and is associated with chronic inflammatory, axial back pain, oligoarthritis, and enthesitis.^[1,2]^ This disease not only manifests itself with joint problems but also with extraarticular problems such as uveitis, bone, kidney, and skin involvement, and intestinal, lung, and heart diseases.^[3]^ Previous reports have revealed that the prevalence of cardiac involvement in patients with AS is twice as high as in the general population.^[4]^ Since AS is a chronic, low-grade inflammatory course, subclinical atherosclerosis may develop in AS patients even in the absence of classical risk factors. Due to their pro- and antiinflammatory properties, macrophages are at the intersection of the basic pathogenetic processes of AS and atherosclerosis.^[5–7]^

Syndecan-4 (SDC4) is a member of the syndecan family of major cell surface heparan sulfate proteoglycan receptors widely expressed in several tissues. This receptor has a protein core and is covalently bound by vertilinear polysaccharide chains. SDC4 mediates a variety of biological processes, including cell migration, proliferation, adhesion, endocytosis, tissue repair and regeneration, cell–matrix interactions, and matrix remodeling.^[8–11]^ Several studies have shown that stress factors, such as ischemia, hypoxia, and infection, can trigger the expression of SDC4 receptors.^[12,13]^ It is well known that macrophage-mediated vascular inflammatory responses play a crucial role in the formation of atherosclerosis.^[14–16]^ Yet, the role of SDC4 in macrophage-mediated atherogenesis in AS patients remains unclear. Therefore, this study investigated the role of SDC4 in subclinical atherosclerosis in AS patients.

## 2. Methods

### 2.1. Study population

In this study, subjects were selected from patients with AS who applied to the Necmettin Erbakan University Meram Medical Faculty Hospital Rheumatology outpatient clinics between January 2020 and December 2022. The control group was selected from people who applied to the cardiology outpatient clinic with nonspecific symptoms and did not have any additional diseases. The diagnosis of AS was made according to the Modified New York and American College of Rheumatology criteria.^[17]^ Patients with a prior history of coronary artery disease, severe valvular heart disease, congestive heart failure, atrial fibrillation, malignancy, renal failure, pregnancy, autoimmune diseases, and those under 18 years of age were excluded from the study. After applying exclusion criteria, eligible patients were included in the study as a study group. All participants’ past medical records and clinical and demographic characteristics were scanned and underwent comprehensive physical examination. In addition, carotid intima-media thickness (CIMT) measurement and disease activity index (Bath Ankylosing Spondylitis Disease Activity Index [BASDAI] measurement) were applied to all patients. BASDAI was calculated by rheumatology specialist physicians. Informed consent of all participants was taken, and approval was obtained from the local ethics committee of our hospital.

### 2.2. Data collection

The patient demographics, comorbidities, medications, and laboratory parameters were obtained from patient records and the hospital database. A comprehensive metabolic panel was performed to measure full blood count, liver and kidney functions, and lipid concentrations. Blood specimens were collected from the antecubital vein after 12-hour fasting before the data collection. All metabolic panel measurements were performed using an autoanalyzer (Roche Diagnostic Modular Systems, Tokyo, Japan). Syndecan-4 levels were analyzed by enzyme-linked immunosorbent assay (ELISA) using a commercial kit (IBL International, Hamburg, Germany) according to factory instructions. CIMT was measured using a Siemens Acuson S3000 ultrasound device using a 9L4 (4.0–9.0 MHz) linear transducer. The subjects’ carotid system was evaluated in B-mode, pulsed Doppler mode, and color mode, with the subject in the supine position, with their head slightly turned to the contralateral side of the carotid artery being examined. Carotid IMT is measured by calculating the space between the intimal-luminal and medial-adventitial interfaces of the carotid artery. In our study, the IMT average of both common carotid arteries was taken.

### 2.3. Statistical analysis

All statistical analyses were conducted using SPSS software version 24.0 for Windows (SPSS Inc., Chicago, IL, USA). Continuous data are presented as mean ± standard deviation (SD) if normally distributed, or as median (25th–75th percentiles), if not normally distributed. Continuous variables were compared using Student’s t test or the Mann–Whitney *U* test. Categorical variables are expressed as numbers and percentages and were analyzed using the χ^2^ test or Fisher exact test. The effect of various variables on AS was calculated by univariate regression analysis. In these analyses, variants with unadjusted *P* < .1 were determined as confounding factors and were included in multivariate regression analyses to determine the independent predictors of AS. Spearman correlation analysis was used to identify factors associated with CIMT in the patient group. Linear regression analysis was performed to identify independent associates of CIMT in the patient group. A 2-tailed *P* < .05 was considered statistically significant during the study.

## 3. Results

Between January 2020 and December 2022, 48 AS patients (study group) who met the study criteria were included in the study. In the same period, 48 people were included in the study as age- and sex-matched control group. The baseline demographic, clinical, and laboratory characteristics of both groups are shown in Table 1. There was no statistically significant difference between the 2 groups in terms of clinical characteristics (*P* > .05). Regarding baseline laboratory values, both groups had similar laboratory properties (*P* > .05). Yet, serum C-reactive protein (CRP) levels were significantly higher among AS patients compared with the control group (5.2 [0.3–74.0] mg/L vs 1.4 [0.1–11.1] mg/L, *P* < .05). Serum SDC4 level was also significantly higher among AS patients compared with the control group (6.7 [1.5–35.0] ng/mL vs 5.1 [0.1–12.5] ng/mL, *P* < .001). With respect to carotid system ultrasound findings, the calculated CIMT was significantly higher in AS patients than in the control group (0.6 [0.3–0.9] mm vs 0.4 [0.2–0.7], *P* < .001).

**Table 1:** Baseline parameters of the study population (n: 96).

Parameters	Patient group (n: 48)	Control group (n:48)	*P*
Age (y)	39 (20–66)	41 (22–65)	.592
Gender (males, n [%])	33 (68.8)	33 (68.8)	1.000
Heart rate (/min)	73.5 (50–100)	70.5 (55–102)	.369
WBC count (×10^6^/µL)	7.6 (4.4–13.1)	8.1 (3.8–11.1)	.680
Hemoglobin (g/dL)	14.6 (10.2–17.3)	14.4 (10.5–18.7)	.378
Platelet count (×10^3^/µL)	286 (143–508)	303 (151–523)	.942
Serum creatinine (mg/dL)	0.84 (0.45–1.25)	0.80 (0.56–1.10)	.766
Alanine aminotransferase (U/L)	17.6 (10.5–36.0)	17.4 (10.5–29.6)	.555
LDL (mg/dL)	96 (41–175)	104 (40–169)	.360
HDL (mg/dL)	44 (30–135)	50 (24–109)	.459
CRP (mg/L)	5.2 (0.3–74.0)	1.4 (0.1–11.1)	.007*
Syndecan (ng/ml)	6.7 (1.5–35.0)	5.1 (0.1–12.5)	<.001*
CIMT (mm)	0.06 (0.03–0.09)	0.04 (0.02–0.07)	<.001*

CIMT = carotid intima-media thickness, CRP = C-reactive protein, HDL = high density lipoprotein, LDL = low density lipoprotein, WBC = white blood cell.

* Indicates statistically significant.

In addition, several variables were included in the univariate Cox regression analysis to demonstrate the relationship between baseline features and the presence of AS. After removing the variables that do not affect the presence of AS in univariate analysis, Cox multivariate regression analysis was performed, that identified serum CRP level and serum SDC4 level as the independent predictors of AS (Table 2). There was no relationship between BASDAI and SDC4. These 2 parameters, together with the calculated BASDAI scores were found to be independent associates of CIMT according to Spearman correlation analysis. According to linear regression analysis, serum SDC4 level was the best predictor of CIMT among the aforementioned parameters (*P* = .004; Table 3).

**Table 2:** Univariate and multivariate binomial regression analyses demonstrating the relationship between baseline characteristics and presence of ankylosing spondylitis.

Parameters	Univariate analysis			Multivariate analysis		
	OR	95% CI	*P*	OR	95% CI	*P*
CRP (mg/L)	1.116	1.029–1.210	.008*	1.106	1.012–1.208	.026*
Syndecan (ng/mL)	1.365	1.108–1.682	.003*	1.399	1.099–1.781	.006*

CRP = C-reactive protein.

* Indicates statistically significant.

**Table 3:** Correlation and linear regression analysis between CIMT and baseline characteristics in the ankylosing spondylitis patients.

Parameters	Spearman correlation analysis		Multivariate linear regression analysis		
	r	*P*	B ± S.E.	95% CI	*P*
Age (y)	0.367	.010*	0.000 ± 0.000	0.000–0.001	.101
Heart rate (/min)	0.057	.699			
WBC count (×106/µL)	0.125	.398			
Hemoglobin (g/dL)	–0.100	.502			
Platelet count (×103/µL)	0.176	.253			
Serum creatinine (mg/dL)	–0.096	.516			
Alanine aminotransferase (U/L)	0.023	.876			
LDL (mg/dL)	–0.040	.815			
HDL (mg/dL)	0.062	.744			
CRP (mg/L)	0.320	.032*	0.000 ± 0.000	0.000–0.000	.500
Syndecan (ng/mL)	0.470	.001*	0.001 ± 0.000	0.000–0.001	.004*
BASDAI	0.354	.016*	0.001 ± 0.001	0.000–0.002	.206

BASDAI = Bath Ankylosing Spondylitis Disease Activity Index, CIMT = carotid intima-media thickness, CRP = C-reactive protein, HDL = high density lipoprotein, LDL = low density lipoprotein, WBC = white blood cell.

* Indicates statistically significant.

## 4. Discussion

Despite advances in the diagnosis and treatment of AS, the risk of cardiovascular complications in AS patients is still higher than in the general population.^[18,19]^ As with other autoimmune rheumatic diseases, cardiovascular events in AS patients mostly result from the early development and rapid progression of atherosclerotic coronary lesions.^[20–23]^ Previous reports revealed that cardiovascular complications develop in AS patients with low or moderate cardiovascular risk, but with high clinical activity of the disease. Furthermore, a direct link has been demonstrated between the progression of subclinical carotid atherosclerosis and the clinical activity of autoimmune rheumatic diseases.^[24]^ Although many factors contribute to the development of cardiovascular complications in AS, it is still unknown which factors play an important role in the development of atherosclerosis in AS patients. However, recent studies have highlighted the potential role of macrophage dysfunction in the inflammatory mechanisms of various autoimmune rheumatic diseases and atherosclerosis. According to these studies, macrophage-mediated expression of proinflammatory molecules reduces new collagen synthesis and matrix metalloproteinases resulting in endothelial dysfunction, increased arterial stiffness and plaque remodeling, and ultimately vulnerable plaque rupture.^[25–27]^

In our study, we investigated the relationship between subclinical atherosclerosis and disease activity using a novel biomarker known as SDC4. In terms of the detection of subclinical atherosclerosis, CIMT was used, whose reliability has been proven by many studies. According to our data, serum SDC4 level and calculated CIMT were significantly higher in AS patients compared with the control group (*P* < .001). The outcomes of our study comply with the result of a meta-analysis conducted by Yuan et al^[28]^ that revealed calculated CIMT was significantly higher in patients with AS compared with healthy controls (standardized mean differences = 0.725, 95% CI = 0.443–1.008, *P* < .001). It has been shown in large-scale studies that CIMT measurements are directly affected by glucose levels, TSH levels, smoking and alcohol consumption, and this issue should also be taken into consideration in the evaluation. Moreover, studies conducted by Gonzalez-Juanatey et al^[29]^ and Rueda-Gotor et al^[30]^ revealed a higher prevalence of carotid plaques in patients with AS compared with healthy controls. Although intima-media thickening was found to be strongly associated with increased arterial stiffness, endothelial dysfunction, and the atherosclerosis process in these studies, the exact mechanism was unclear. They postulated that genetic factors and persistent systemic inflammation work together in the process of atherosclerosis.^[29,30]^ Based on these data, we used SDC4, which is an important indicator of increased systemic inflammatory process.

SDC4, a heparan sulfate proteoglycan (HSPG), is widely expressed in endothelial and epithelial cells. It can cooperate with various receptors and play regulatory roles in different processes, including wound healing, inflammation, and atherosclerosis.^[31,32]^ SDC4 increases mineralization in vascular smooth muscle cells, which are important in atherosclerotic plaques.^[33]^ SDC1, in the same group as SDC4, an HSPG that uses different intracellular transduction mechanisms, was recently discovered to correlate with subclinical atherosclerosis in AS patients.^[33]^ In animal experiments, different results were found regarding the relationship between SDC4 and atherosclerosis. Hu et al^[34]^ found that blocking SDC4 degranulation slowed atherosclerosis in mice. Zimmer et al^[33]^ showed that inhibition of SDC4-mediated signaling pathways reduces vascular oxidative stress, improves endothelial function, and attenuates atherogenesis. Moreover, increased SDC4 expression in repaired areas of injured cardiac tissues has been interpreted as overexpression of SDC4 under ischemic conditions.^[35]^ Therefore, increased serum SDC4 levels are accepted as an indicator of the increase in the atherosclerotic process as well as the inflammatory process. In our study, we observed a positive correlation between serum SDC4 level and calculated CIMT in AS patients. This result indicates that SDC4 is likely to be a marker of subclinical atherosclerosis in AS patients and can be used as an accurate marker in determining the risk of cardiovascular complications. In addition, the results of our study can be interpreted in favor of a strong association between increased SDC4 expression and higher inflammatory activity, as well as an increased risk of cardiovascular complications.

## 5. Limitations

Our study has some limitations. First, it was a single-center study based on relatively small sample size. Second, serum SDC4 levels were calculated as a single measurement and not as repeated measurements, which may hinder the observation of long-term effects. Third, we did not compare the measurement of the SDC4 level with other well-known proinflammatory biomarkers for predicting subclinical atherosclerosis. Last but not least, the fact that our study sample consisted of patients who applied to our outpatient clinic limits the generalizability of the results.

## 6. Conclusion

Our data show that serum SDC4 levels provide comprehensive information about the clinical activity of the disease and subclinical atherosclerosis in AS patients. Due to the limited data on the effects of antirheumatic treatment options and the determination of cardiovascular risk factors associated with AS, it is very important to identify risky patients who require early diagnosis and close follow-up. In this context, SDC4 is likely to be a suitable biomarker for the early prediction of subclinical atherosclerosis in AS patients.

## Author contributions

**Conceptualization:** Ahmet Lütfü Sertdemir, Yakup Alsancak.

**Data curation:** Ahmet Lütfü Sertdemir, İrem Oktay.

**Formal analysis:** Mustafa Duran.

**Investigation:** Mustafa Duran, Yakup Alsancak.

**Methodology:** Mustafa Çelik.

**Project administration:** Ahmet Taha Şahin.

**Resources:** Mustafa Çelik.

**Software:** Sefa Tatar.

**Supervision:** Ahmet Taha Şahin, Yakup Alsancak.

**Validation:** Sefa Tatar.

**Visualization:** İrem Oktay.

**Writing—original draft:** Ahmet Lütfü Sertdemir, Ahmet Taha Şahin.

## References

[1] Reveille JD Arnett FC. Spondyloarthritis: update on pathogenesis and management. Am J Med. 2005;118:592–603. 15922688 10.1016/j.amjmed.2005.01.001

[2] Khan MA. Update on spondyloarthropathies. Ann Intern Med. 2002;136:896–907. 12069564 10.7326/0003-4819-136-12-200206180-00011

[3] El Maghraoui A. Extra-articular manifestations of ankylosing spondylitis: prevalence, characteristics and therapeutic implications. Eur J Intern Med. 2011;22:554–60. 22075279 10.1016/j.ejim.2011.06.006

[4] Haroon NN Paterson JM Li P. Patients with ankylosing spondylitis have increased cardiovascular and cerebrovascular mortality. Ann Intern Med. 2015;163:409–16. 26258401 10.7326/M14-2470

[5] Balčiūnaitė A Budrikis A Rumbinaitė E. Ankylosing spondyloarthritis resulting severe aortic insufficiency and aortitis: exacerbation of ankylosing spondyloarthritis and stenosis of the main left coronary artery after mechanical aortic valve implantation with cardiopulmonary bypass. Case Rep Rheumatol. 2020;2020:9538527. 31984147 10.1155/2020/9538527 PMC6964718

[6] Fernández-Alvarez V Linares Sánchez M López Alvarez F. Evaluation of intima-media thickness and arterial stiffness as early ultrasound biomarkers of carotid artery atherosclerosis. Cardiol Ther. 2022;11:231–47. 35362868 10.1007/s40119-022-00261-x PMC9135926

[7] Addi C Presle A Fremont S. The Flemmingsome reveals an ESCRT-to-membrane coupling via ALIX/syntenin/syndecan-4 required for completion of cytokinesis. Nat Commun. 2020;11:1941. 32321914 10.1038/s41467-020-15205-z PMC7176721

[8] Valdivia A Cardenas A Brenet M. Syndecan-4/PAR-3 signaling regulates focal adhesion dynamics in mesenchymal cells. Cell Commun Signal. 2020;18:129. 32811537 10.1186/s12964-020-00629-3 PMC7433185

[9] Bellin RM Kubicek JD Frigault MJ. Defining the role of syndecan-4 in mechanotransduction using surface-modification approaches. Proc Natl Acad Sci U S A. 2009;106:22102–7. 20080785 10.1073/pnas.0902639106 PMC2796905

[10] Herum KM Lunde IG Skrbic B. Syndecan-4 is a key determinant of collagen cross-linking and passive myocardial stiffness in the pressure-overloaded heart. Cardiovasc Res. 2015;106:217–26. 25587045 10.1093/cvr/cvv002

[11] Ozsoy HZ Sivasubramanian N Wieder ED. Oxidative stress promotes ligand-independent and enhanced ligand-dependent tumor necrosis factor receptor signaling. J Biol Chem. 2008;283:23419–28. 18544535 10.1074/jbc.M802967200 PMC2516989

[12] Julien MA Wang P Haller CA. Mechanical strain regulates syndecan-4 expression and shedding in smooth muscle cells through differential activation of MAP kinase signaling pathways. Am J Physiol Cell Physiol. 2007;292:C517–525. 16822948 10.1152/ajpcell.00093.2006

[13] Koelwyn GJ Corr EM Erbay E. Regulation of macrophage immunometabolism in atherosclerosis. Nat Immunol. 2018;19:526–37. 29777212 10.1038/s41590-018-0113-3 PMC6314674

[14] Tabas I Bornfeldt KE. Macrophage phenotype and function in different stages of atherosclerosis. Circ Res. 2016;118:653–67. 26892964 10.1161/CIRCRESAHA.115.306256 PMC4762068

[15] Martinez GJ Celermajer DS Patel S. The NLRP3 inflammasome and the emerging role of colchicine to inhibit atherosclerosis-associated inflammation. Atherosclerosis. 2018;269:262–71. 29352570 10.1016/j.atherosclerosis.2017.12.027

[16] Ward MM Deodhar A Gensler LS. Update of the American college of rheumatology/spondylitis association of America/spondyloarthritis research and treatment network recommendations for the treatment of ankylosing spondylitis and nonradiographic axial spondyloarthritis. Arthritis Rheumatol. 2019;71:1599–613. 31436036 10.1002/art.41042 PMC6764882

[17] Agca R Heslinga SC van Halm VP. Atherosclerotic cardiovascular disease in patients with chronic inflammatory joint disorders. Heart. 2016;102:790–5. 26888573 10.1136/heartjnl-2015-307838

[18] Van den Hoek J Boshuizen HC Roorda LD. Mortality in patients with rheumatoid arthritis: a 15-year prospective cohort study. Rheumatol Int. 2017;37:487–93. 28032180 10.1007/s00296-016-3638-5 PMC5357293

[19] Mahtta D Gupta A Ramsey DJ. Autoimmune rheumatic diseases and premature atherosclerotic cardiovascular disease: an analysis from the VITAL registry. Am J Med. 2020;133:1424–1432.e1. 32598903 10.1016/j.amjmed.2020.05.026

[20] Hedar AM Stradner MH Roessler A. Autoimmune rheumatic diseases and vascular function: the concept of autoimmune atherosclerosis. J Clin Med. 2021;10:4427. 34640445 10.3390/jcm10194427 PMC8509415

[21] Demir K Avci A Ergulu Esmen S. Assessment of arterial stiffness and epicardial adipose tissue thickness in predicting the subclinical atherosclerosis in patients with ankylosing spondylitis. Clin Exp Hypertens. 2021;43:169–74. 33028113 10.1080/10641963.2020.1833025

[22] Atzeni F Nucera V Galloway J. Cardiovascular risk in ankylosing spondylitis and the effect of anti-TNF drugs: a narrative review. Expert Opin Biol Ther. 2020;20:517–24. 31847607 10.1080/14712598.2020.1704727

[23] Kocabaş V. The level of serum oxidant-antioxidant in patients with ankylosing spondylitis. Selcuk Med J. 2010;26:79–83.

[24] Dalbeni A Giollo A Bevilacqua M. Traditional cardiovascular risk factors and residual disease activity are associated with atherosclerosis progression in rheumatoid arthritis patients. Hypertens Res. 2020;43:922–8. 32341443 10.1038/s41440-020-0441-1

[25] Gerasimova EV Popkova TV Gerasimova DA. Macrophage dysfunction in autoimmune rheumatic diseases and atherosclerosis. Int J Mol Sci. 2022;23:4513. 35562903 10.3390/ijms23094513 PMC9102949

[26] Lin J Kakkar V Lu X. Impact of matrix metalloproteinases on atherosclerosis. Curr Drug Targets. 2014;15:442–53. 24517161 10.2174/1389450115666140211115805

[27] Hreggvidsdottir HS Noordenbos T Baeten DL. Inflammatory pathways in spondyloarthritis. Mol Immunol. 2014;57:28–37. 23969080 10.1016/j.molimm.2013.07.016

[28] Yuan Y Yang J Zhang X. Carotid intima-media thickness in patients with ankylosing spondylitis: a systematic review and updated meta-analysis. J Atheroscler Thromb. 2019;26:260–71. 30089757 10.5551/jat.45294 PMC6402883

[29] Gonzalez-Juanatey C Vazquez-Rodriguez TR Miranda-Filloy JA. The high prevalence of subclinical atherosclerosis in patients with ankylosing spondylitis without clinically evident cardiovascular disease. Medicine (Baltim). 2009;88:358–65. 10.1097/MD.0b013e3181c10773 19910750

[30] Rueda-Gotor J Corrales A Blanco R. Atherosclerotic disease in axial spondyloarthritis: increased frequency of carotid plaques. Clin Exp Rheumatol. 2015;33:315–20. 26005760

[31] Vuong TT Reine TM Sudworth A. Syndecan-4 is a major Syndecan in primary human endothelial cells in vitro, modulated by inflammatory stimuli and involved in wound healing. J Histochem Cytochem. 2015;63:280–92. 25575567 10.1369/0022155415568995 PMC4374057

[32] Xie J Wang J Li R. Syndecan-4 over-expression preserves cardiac function in a rat model of myocardial infarction. J Mol Cell Cardiol. 2012;53:250–8. 22561100 10.1016/j.yjmcc.2012.04.014

[33] Zimmer S Goody PR Oelze M. Inhibition of Rac1 GTPase decreases vascular oxidative stress, improves endothelial function, and attenuates atherosclerosis development in mice. Front Cardiovasc Med. 2021;8:680775. 34422919 10.3389/fcvm.2021.680775 PMC8377253

[34] Hu X Chen J Tang W. Effects of exercise programs on pain, disease activity and function in ankylosing spondylitis: a meta‐analysis of randomized controlled trials. Eur J Clin Invest. 2020;50:e13352. 32683694 10.1111/eci.13352

[35] Kojima T Takagi A Maeda M. Plasma levels of syndecan-4 (ryudocan) are elevated in patients with acute myocardial infarction. Thromb Haemost. 2001;85:793–9. 11372670

